# Demographic responses of hybridizing cinquefoils to changing climate in the Colorado Rocky Mountains

**DOI:** 10.1002/ece3.10097

**Published:** 2023-07-11

**Authors:** Kelly A. Carscadden, Daniel F. Doak, Meagan F. Oldfather, Nancy C. Emery

**Affiliations:** ^1^ Department of Ecology and Evolutionary Biology University of Colorado Boulder Boulder Colorado USA; ^2^ Department of Environmental Studies University of Colorado Boulder Boulder Colorado USA

**Keywords:** climate change, demography, evolutionary ecology, hybridization, integral projection models (IPMs), montane plants, population modelling, potentilla

## Abstract

Hybridization between taxa generates new pools of genetic variation that can lead to different environmental responses and demographic trajectories over time than seen in parental lineages. The potential for hybrids to have novel environmental tolerances may be increasingly important in mountainous regions, which are rapidly warming and drying due to climate change. Demographic analysis makes it possible to quantify within‐ and among‐species responses to variation in climate and to predict population growth rates as those conditions change. We estimated vital rates and population growth in 13 natural populations of two cinquefoil taxa (*Potentilla hippiana* and *P. pulcherrima*) and their hybrid across elevation gradients in the Southern Rockies. Using three consecutive years of environmental and demographic data, we compared the demographic responses of hybrid and parental taxa to environmental variation across space and time. All three taxa had lower predicted population growth rates under warm, dry conditions. However, the magnitude of these responses varied among taxa and populations. Hybrids had consistently lower predicted population growth rates than *P. hippiana*. In contrast, hybrid performance relative to *P. pulcherrima* varied with population and climate, with the hybrid maintaining relatively stable growth rates while populations of *P. pulcherrima* shrank under warm, dry conditions. Our findings demonstrate that hybrids in this system are neither intrinsically unfit nor universally more vigorous than parents, suggesting that the demographic consequences of hybridization are context‐dependent. Our results also imply that shifts to warmer and drier conditions could have particularly negative repercussions for *P. pulcherrima*, which is currently the most abundant taxon in the study area, possibly as a legacy of more favorable historical climates. More broadly, the distributions of these long‐lived taxa are lagging behind their demographic trajectories, such that the currently less common *P. hippiana* could become the most abundant of the *Potentilla* taxa as this region continues to warm and dry.

## Graphical abstract

Using multiple populations of two cinquefoil taxa and their naturally occurring hybrid, we compare the demographic trajectories of these close relatives under changing environments. Our analyses suggest that the distributions of these long‐lived taxa are lagging behind their demographic responses to environmental change.

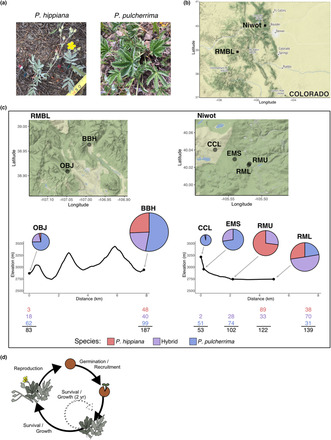

## 1 INTRODUCTION

Hybridization (interbreeding between taxa) is common in plants (Mallet, 2005) and alters the pool of genetic variation available for population responses to changing environments (Lexer, Randell, & Rieseberg, 2003). There are three general hypotheses for how genetic variation generated through hybridization will influence individual fitness. Two hypotheses focus on genetic (intrinsic) factors: enhanced genetic variation may cause hybrids to have higher fitness than their parents across environments (“hybrid vigor” or “heterosis”; for example, East, 1936; Huang et al., 2015), or, conversely, genetic incompatibilities between parent genomes or the disruption of coadapted gene complexes could depress hybrid fitness relative to their parent taxa in any environment (Mayr, 1942). A third hypothesis contends that extrinsic factors (selective environments) impact the relative performance of hybrids and parents (e.g., “bounded hybrid superiority”; Abbott & Brennan, 2014; Freeman et al., 1999; Fritz et al., 2006). For instance, hybrids can have intermediate or transgressive traits (more extreme than either parent taxon) that are maladaptive in parent habitats but favorable in other environments (Lexer, Welch, et al., 2003). Despite the widespread recognition of hybridization as an important source of genetic and phenotypic variation in plant evolution (Ellstrand & Schierenbeck, 2000; Soltis & Soltis, 2009), the population dynamics and environmental tolerances of hybrid plant lineages have seldom been characterized in the field (Vilà et al., 2000).

The distributions of plant species largely hinge upon the interaction between environmental variation (across a landscape and over time) and genetic variation within and among populations (Bemmels & Anderson, 2019; Carscadden et al., 2020). Hybridization provides ‘natural experiments’ to determine how genetic and environmental sources of variation structure species' environmental tolerances. Several lines of evidence indicate that hybrids (compared to parent lineages) may better withstand novel or extreme environments that emerge through climate change. Hybrids have been found to tolerate a wider range of environments than parent lineages (e.g., a transplant study in *Iris* reported hybrids outperforming parent taxa across parent and hybrid habitats; Emms & Arnold, 1997). An experiment in *Ipomopsis* showed that hybrid (but not parent) floral production increased with drought treatments (Campbell & Wendlandt, 2013). Additionally, hybrids with transgressive traits have been shown to tolerate extreme environments that could become more prevalent with climate change (e.g., *Helianthus* hybrids adapted to salt marshes; Lexer, Welch, et al., 2003). However, hybrid performance (relative to parents) under novel conditions may depend on the environments in which they evolve (Shukla et al., 2021), and it is unclear how these insights on aspects of hybrid performance translate into population growth of hybrids across environments.

Demographic models provide a quantitative framework for evaluating the collective fates of genes, individuals, and populations and are, therefore, ideal for addressing questions at the nexus of ecology and evolution (Metcalf & Pavard, 2007). Whether populations of different species have similar or contrasting demographic trajectories under changing environments will also influence how climate change impacts assemblages of species. Population growth rates are calculated by combining estimates of vital rates across the lifecycle (e.g., survival, germination, recruitment, growth, and reproduction; Morris & Doak, 2002), generating a measure of population responses to environmental variation that integrates across vital rates and potentially varying responses of different life stages (Doak & Morris, 2010; Laughlin et al., 2020; Pironon et al., 2018; Sheth & Angert, 2018). Demographic approaches are particularly informative for long‐lived organisms whose local numbers or extinction rates can lag behind the pace of environmental change, such that species survive in areas that are no longer capable of supporting self‐sustaining populations (Pagel et al., 2020). The potential decoupling of abundance and habitat suitability makes it important to estimate population growth rates to understand how species are responding to changing conditions (Ehrlén & Morris, 2015; Laughlin et al., 2020).

To determine how interbreeding taxa respond to environmental variation in space and time, we used demographic data to estimate population growth rates in two plant taxa and their naturally occurring hybrid in a mountain landscape. Mountainous areas are spatially and temporally heterogeneous environments that are rapidly changing with global warming (Gottfried et al., 2012; Pepin et al., 2015; Thuiller et al., 2005; Williams et al., 2015). We studied perennial cinquefoils in the genus *Potentilla* (Rosaceae), which are prevalent in mountainous regions (Dobes & Paule, 2010) and are known for high rates of hybridization that generate “a maddening array of shapes and forms” (Ackerfield, 2015). We focused on two relatively common and widespread taxa in the Southern Rocky Mountains: *Potentilla hippiana*, *Potentilla pulcherrima*, and their hybrid. In this region, *P. pulcherrima* is particularly abundant, especially in wet meadows, while *P. hippiana* is less common and primarily found on rocky inclines, dry meadows, or disturbed patches (Carscadden, personal observation). Habitat affinities of hybrid populations are less well known but appear intermediate (Carscadden, personal observation). Here we test whether population growth rates will differ across environmental gradients and among our two focal taxa and their hybrids. Specifically, if current distributions reflect demographic responses to environments (rather than lags), we expect that *P. hippiana* population growth will be confined to dry conditions and *P. pulcherrima* will maintain growing populations across a broader range of environments. Alternatively, if there is a mismatch between current conditions and the occurrence and abundance of these taxa (Duchenne et al., 2021; Nomoto & Alexander, 2021), populations could be declining under conditions where mature plants are still present. Hybrid populations are relatively common, which could suggest that they are not intrinsically unfit, that they are sink populations sustained by ongoing hybridization, or that hybrids are favored under some conditions but not others. By quantifying growth rates of multiple populations of hybridizing taxa across natural environmental gradients, we aimed to differentiate among these contrasting explanations for montane plant distributions and better understand within‐ and among taxa responses to climate variation.

## 2 MATERIALS AND METHODS

### 2.1 Study system

**FIGURE 1:**
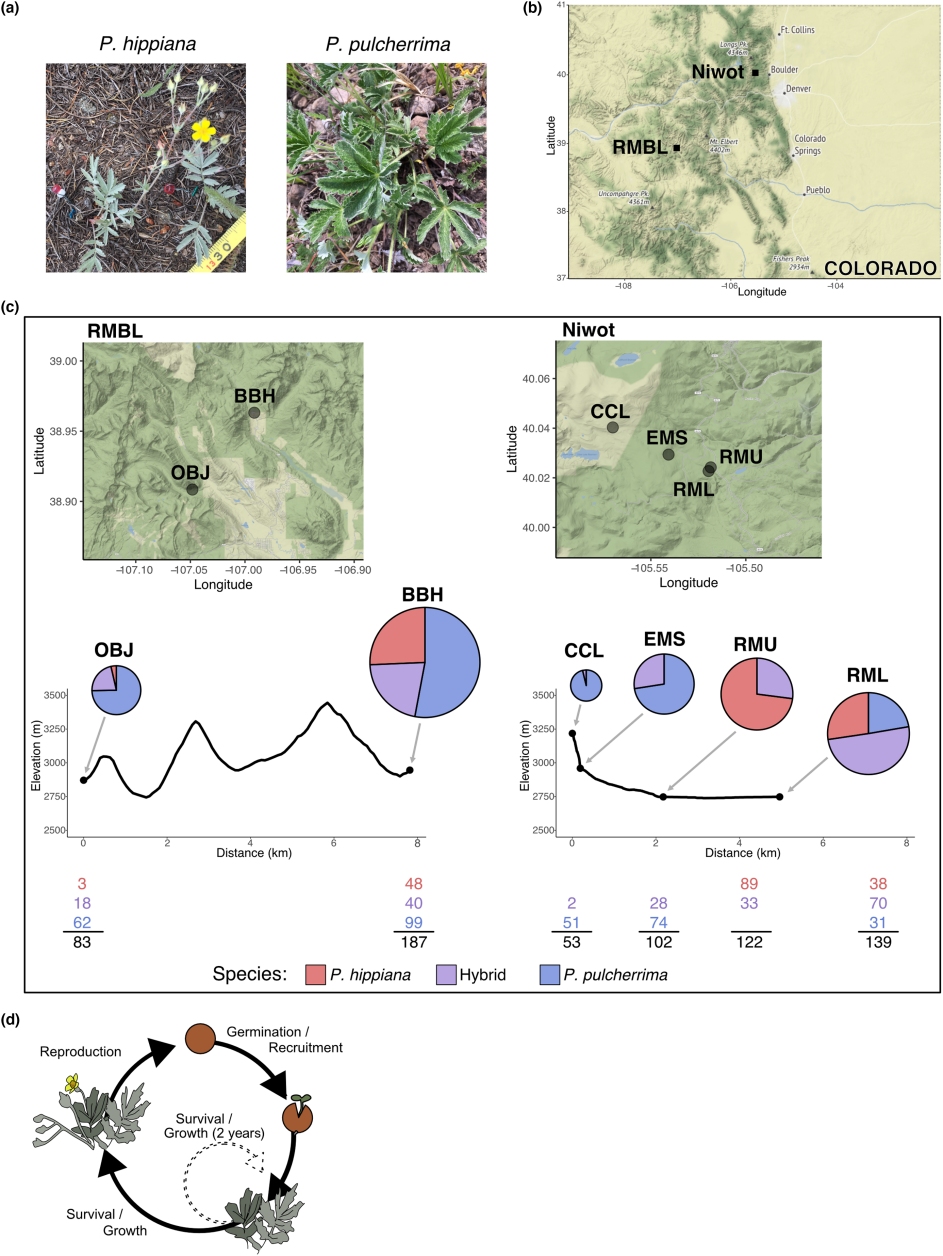
Study system and sites. (a) *Potentilla* parent taxa. (b) Study regions across the Continental Divide in Colorado: Rocky Mountain Biological Laboratory (RMBL) and Niwot Ridge LTER. (c) Demographic sites near RMBL and Niwot Ridge. Lower panels show elevation profiles. Pie charts show relative proportions of each taxon at each site in 2020. The pie chart size is scaled by total individuals per site; 2020 counts of tagged plants per taxon are included below. (d) Life cycle schematic for example *Potentilla* taxon.

**FIGURE A1:**
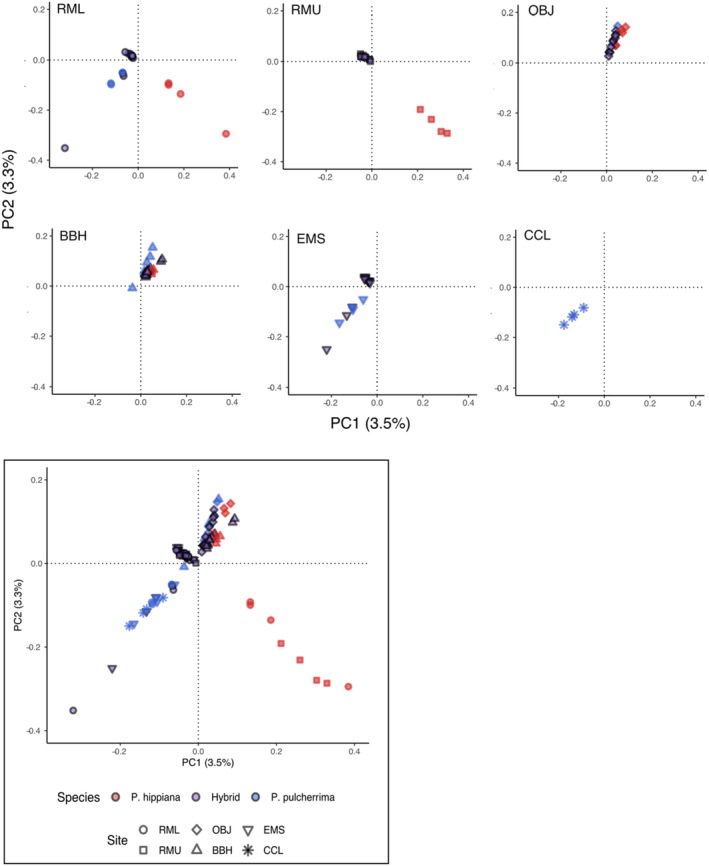
PCA of RADseq data of parent and hybrid *Potentilla*. Sequence data is shown by site (panels and shapes) and taxon (colors; hybrids are denoted in purple with black outlines). Note that the two RMBL sites (OBJ, BBH) include *P. hippiana* var *hippiana*, while Niwot Ridge sites include *P. hippiana* var *effusa* (Ackerfield, 2015). At each site, we sampled 2–5 individuals of each parent taxon that was present and 10–11 individuals that we had identified as hybrids based on morphology. Leaflets of tagged individuals (from the demographic study – see Section 2) were collected on silica gel, freeze‐dried, homogenized, and DNA extracted using DNeasy plant mini kits (Qiagen). A double‐digest RADseq library using *EcoR1* and *Mse1* restriction enzymes was prepared and sequenced following (Beeler et al., 2020; Tripp et al., 2017). Blue pippin size selection and sequencing were performed at the Biofrontiers Sequencing Facility at the University of Colorado. We used iPYRAD to filter, clean, and demultiplex the data (Beeler et al., 2020; Eaton & Overcast, 2020) and we performed a Principal Components Analysis using the Variant Call Format (VCF) table output to examine sequence variation among parent and putative hybrid taxa.

*Potentilla* is especially well‐suited to addressing questions about variation in perennial plant phenotypes and performance across steep environmental gradients (Clausen et al., 1940). Both *P. hippiana* and *P. pulcherrima* are herbaceous, rosette perennials that are morphologically distinct (Figure 1a): *P. hippiana* has pinnate leaves while *P. pulcherrima* leaves are palmately arranged. Hybrids between these parent taxa are a named group that is described based on morphological characteristics in the *Potentilla* taxonomic key (Ackerfield, 2015) as similar in size to *P. hippiana* (or shorter than the average *P. pulcherrima*) with subdigitate leaves. These focal taxa occur across a range of elevations on the eastern and western slopes of the Continental Divide (*P. hippiana* from 6000 to 11,500 ft [1829–3505 m] and *P. pulcherrima* from 7000 to 13,000 ft [2134–3962 m]; Ackerfield, 2015). The distribution of hybrids has not been described; however, hybrids often co‐occur with parental taxa and appear to occupy intermediate zones between the different parental microhabitats (Carscadden, personal observation). Our genetic data suggest possible gene flow among taxa within sites, as hybrids cluster with parent individuals within sites (vs. hybrids clustering with hybrids across sites; Figure A1 in Appendix A).

### 2.2 Study design

We monitored populations in four subalpine locations east of the Continental Divide near the Niwot Ridge Long‐Term Ecological Research Site (near Nederland, CO) and two sites west of the Continental Divide near the Rocky Mountain Biological Laboratory (RMBL; Figure 1b). We selected sites that differ in the relative abundance of the three *Potentilla* taxa and span the elevations within the subalpine where populations of the focal taxa were most common (Figure 1c).

**FIGURE A2:**
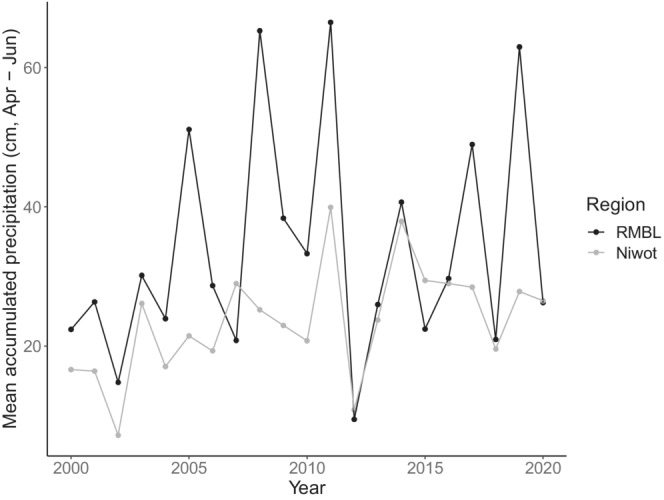
Mean April–June precipitation in the study regions from 2000 to 2020. Data aggregated from SNOTEL stations (Butte and Schofield for RMBL, University Camp and Niwot stations, for Niwot; USDA Natural Resources Conservation Service, 2020). Our demographic study took place in the last 3 years shown, representing the typical inter‐annual moisture fluctuations in each region.

In the summer of 2018, we installed permanently marked, geo‐referenced transects where one or more focal taxon occurred within each site (1–6 transects/site, with more transects in sites with low densities or multiple focal taxa). We established 0.5 × 0.5 m plots at random locations along each transect with a minimum of 0.5 m between adjacent plots. We placed enough transects and/or plots so that at least 50 individuals of the most abundant taxon were sampled at a site, and any apparent gradients in environmental conditions or population density within the transects were included. Additional plots and transects were added in 2019 to increase the sample sizes of relatively undersampled taxa and replace one transect that was lost to avalanche damage. Thus, we had sufficient individuals to estimate demographic rates for 10 populations across the 2018–2019 transition and 13 populations across 2019–2020. We used the taxonomic key for *Potentilla* (Ackerfield, 2015), which includes the hybrid, to identify each individual of our focal taxa in our plots. Individuals of these taxa can spread vegetatively, so we carefully excavated (and then replaced) the soil to ~10 cm depth around the caudex of each stem to identify connected ramets within a ~10 cm radius when necessary to delineate individuals. We tagged individuals with unique combinations of sewing pins and wires with colored beads and recorded the coordinates of each individual within every plot. We monitored ~700 individual plants over the three‐year study period between 2018 and 2020, providing two transitions (2018–2019, 2019–2020) that were used to estimate vital rates for each taxon at each site. The study period captured the inter‐annual environmental variation typical of the Colorado Rockies; long‐term precipitation data at Niwot Ridge and RMBL‐adjacent SNOTEL weather stations indicate that conditions changed from relatively dry to wet (extremely wet at RMBL) between 2018 and 2019, and from wet to average between 2019 and 2020 (Figure A2 in Appendix A).

#### 2.2.1 Measurements

##### Plant traits

Populations were censused in July–August of 2018, 2019, and 2020 during the peak flowering period for *Potentilla*. For each tagged plant, we measured plant vegetative height (stretched length of longest petiole + length of its leaf), the number of leaves in the basal rosette (summed across all connected ramets), reproductive status (flowering or vegetative), and floral production (cumulative buds, flowers, and fruits). We also recorded mortality and marked and measured all new recruits found during the 2019 and 2020 censuses. An aggregate measure of individual size, used as the state variable in subsequent analyses, was calculated as the log‐transformed product of the plant vegetative height and number of basal rosette leaves. We log‐transformed the size measure to capture growth of small plants. This size measure was selected because it performed better than other options (e.g., log height, log basal leaf count) in the following exploratory linear model, which omitted browsed plants: *size*_*t*+1_ ~ *size*_*t*_ × species + site + transition (*R* ^2^ = .77 and residual SE = 0.56).

##### Environmental variables

Vital rates and overall population growth have been found to change dramatically across elevation and environmental gradients (Angert & Schemske, 2005; Clausen et al., 1940). In temperate mountain ranges, warm temperatures early in the growing season can accelerate plant development and reproduction (Dolezal et al., 2020; Stinson, 2004), so seeds can be set before summer drought. High temperatures can dry soils and shorten growing seasons (Jonas et al., 2008), limiting plant survival, growth, or reproduction (Reynolds, 1984; Winkler et al., 2016). The duration of winter snow cover is also key to plant development: areas with long‐lasting snow cover have short growing seasons that constrain plant growth, but snow cover insulates plants from freezing temperatures in the winter and early spring (Jonas et al., 2008; Oldfather & Ackerly, 2019). As snow melts, it provides much of the early‐season moisture that influences plant growth (Litaor et al., 2008).

**FIGURE A3:**
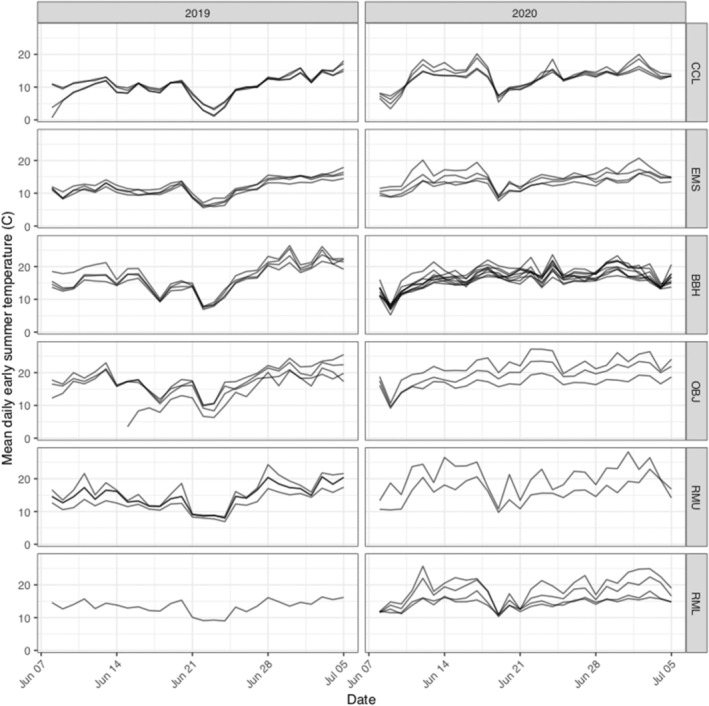
Mean daily early summer soil temperatures. Temperatures are for June 8 to July 5 from iButtons (lines) at transects of demographic sites (rows) in the second and third growing seasons (columns; 2019, 2020). Sites are ordered from low (RML) to high (CCL) elevation. In 2019, one iButton of OBJ was buried by an avalanche and slow to melt out—temperatures were omitted until above 0°C.

**FIGURE A4:**
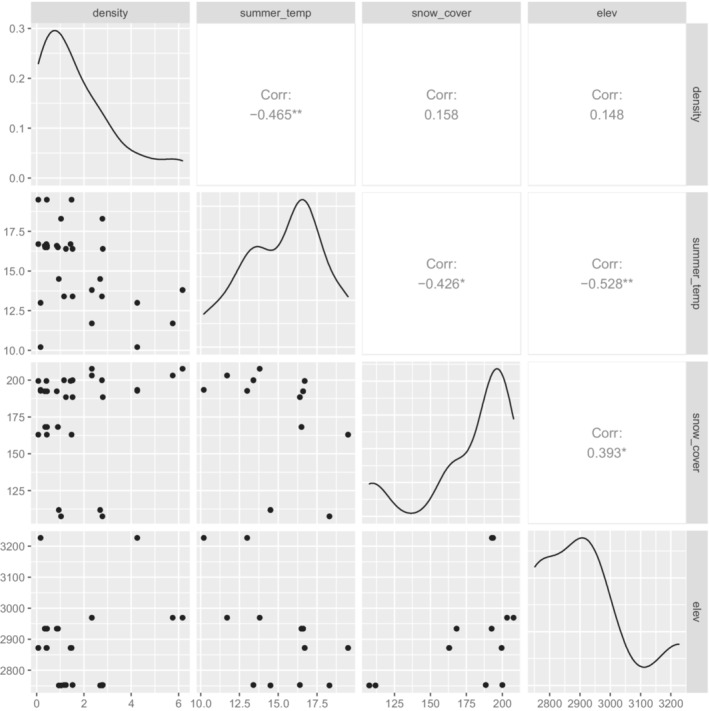
Correlations between continuous predictors. Distributions and relationships between intraspecific density, early summer temperature, days of snow cover, and elevation.

At each site we measured elevation, density of conspecific plants, early summer temperature, and days of winter snow cover. Elevation was recorded at each site using a hand‐held Trimble GPS. We estimated the density of each focal taxon at each site for each year, using our demographic survey data, to account for possible density‐dependence in vital rates (Adler et al., 2018). To quantify growing season temperatures and winter snow cover duration, we used iButton data loggers to record soil temperature every 4.25 h from late summer 2018 through early summer 2020. Each logger was sealed in a small plastic container with silica gel, and a minimum of four loggers per site were buried 2 cm below the soil surface at the ends of transects. Some loggers were lost between surveys, and more loggers were installed at BBH since our plots spanned a larger distance and apparent environmental gradient (from loose rock on a steep slope to a flat, grassy, riverside area) at that site. We extracted daily average soil temperature from each logger (Figure A3 in Appendix A) and calculated site averages for early growing season temperatures, defined as the 4‐week period (June 8 through July 5) preceding each annual census. We excluded 0°C temperatures (including several days in June 2019 from one transect at OBJ due to avalanche activity) to focus on temperatures when plants were growing. We estimated the total days of winter snow cover for each site as the average number of days between September and June in which the daily range of soil temperatures was 1°C or less (Schmid et al., 2012), as snow cover dampens soil temperature fluctuations. Elevation, density, and the climate variables were not strongly collinear (|*r*| < 0.6; Figure A4 in Appendix A).

#### 2.2.2 Integral projection models

Integral Projection Models (IPMs) are one form of size‐structured demographic models, all of which integrate across life‐history transitions to estimate annual population growth (“lambda”, Doak et al., 2021; Easterling et al., 2000; Merow et al., 2014). Our IPMs characterize vital rates (recruitment, survival, growth, and reproduction; Figure 1d) as functions of size and environmental conditions. By modeling the cumulative responses of vital rates to environmental gradients, IPMs can predict population dynamics across a range of environments. All population modeling was conducted in R V.4.0.3 (R Core Team, 2020).

##### Vital rate models

The global model of individual growth predicted plant size at the end of a transition (time *t + 1*) as a function of taxon, starting size (at time *t*), early summer temperature, duration of winter snow cover, population intraspecific density, and pairwise interactions between taxon and each continuous predictor (see Table 1 for global and best model terms). Elevation was not included in the global model for growth because climate data were available for both transitions and climate variables have a clearer mechanistic link to plant performance than elevation, which is a surrogate for many underlying biotic and abiotic gradients.

**TABLE 1:** Vital rate model formulae.

Vital rate (response variable)	Global model	Best model	Error structure
Growth			
*size*_*t*+1_	$\text{taxon}+{\text{size}}_{t}$	+	Gaussian
	${\text{size}}_{t}^{2}$		
	snow cover	+	
	${\text{snow cover}}^{2}$	+	
	summer temp	+	
	${\text{summer temp}}^{2}$		
	density	+	
	${\text{density}}^{2}$	+	
	$\text{taxon}:{\text{size}}_{t}$		
	taxon:snow cover	+	
	taxon:summer temp	+	
	taxon:density	+	
Reproduction			
$\;P\left(\text{flowering}\right)$	$\text{taxon}+{\text{size}}_{t}$	+	Binomial
	${\text{size}}_{t}^{2}$		
	elevation		
	${\text{elevation}}^{2}$		
	density	+	
	${\text{density}}^{2}$	+	
	$\text{taxon}:{\text{size}}_{t}$	+	
	taxon:elevation		
	taxon:density	+	
Floral production	$\text{taxon}+{\text{size}}_{t}$	+	Negative binomial
	${\text{size}}_{t}^{2}$	+	
	elevation	+	
	${\text{elevation}}^{2}$		
	density	+	
	${\text{density}}^{2}$		
	$\text{taxon}:{\text{size}}_{t}$	+	
	taxon:elevation	+	
	taxon:density		
Survival			
$\;P\left(\text{survival}\right)$	$\text{taxon}+{\text{size}}_{t}$	+^a^	Binomial
	${\text{size}}_{t}^{2}$	+	
	$\text{taxon}:{\text{size}}_{t}$		
Recruitment	seedlings/flowers	^b^	

*Note*: Predictors included in global models and those retained (+) in the best models for each vital rate. Continuous predictors were z‐scored. Elevation was not included in the global model for growth because climate data were available for both transitions. We denote interaction terms with ‘:’ following R syntax. Taxon (*taxon*) and ${\text{size}}_{t}$ were included in all models except *P. hippiana* survival (which pooled across taxa) and recruitment (see Section 2), and AICc was used to identify the best combination of additional covariates for each vital rate. The model error structure is given. The negative binomial model was fit using *MASS* (Venables & Ripley, 2002).

^a^ Omitted *taxon* term for pooled model (used for *P. hippiana* survival estimate), so the model would not predict immortality.

^b^ One ratio per taxon per transition.

To characterize annual reproduction, we separately estimated the probability of flowering and counts of flowers produced by reproductive plants (quantified as cumulative buds, flowers, and fruits at the time of census). Floral production was modeled using a negative binomial error structure to handle overdispersion. Whereas plant growth (measured using annual censuses) occurs over transitions, flowering occurs each growing season and would be influenced by that season's climate conditions. Because iButtons were not installed to estimate temperature and snow cover duration before the 2018 census, we instead included elevation as a measure of environmental variation in our global models of reproduction.

Survival probability was modeled without environmental or density variables due to the very low number of observed mortality events and thus weak power to estimate complex models. For *P. pulcherrima* and the hybrid, we kept the coefficients from the best survival model (Table 1). However, since only two *P. hippiana* mortality events occurred in our study, we estimated survival coefficients for this taxon from a model based solely on individual size and using data pooled across taxa.

Recruitment included germination and seedling survival‐to‐census and was estimated as the ratio of the number of seedlings per flower produced in the preceding year. There were few seedlings in the study (11–23 total per taxon across all sites and years), so we estimated a single mean seedling/flower ratio per transition for each taxon by pooling counts across populations. The extremely low seedling/flower ratios observed in the demographic surveys were consistent with a complementary seed transplant experiment we conducted at each site, where we observed zero germination (out of 4000 planted seeds) over 2 years (K. A. Carscadden, N. C. Emery, D. F. Doak, unpublished data).

For global models of individual growth, reproduction, and survival described above, we included linear and quadratic forms of each continuous predictor (size, population intraspecific density, and environmental variables) to allow for nonlinear responses (e.g., Doak & Morris, 2010). All continuous predictors were transformed into z‐scores (scaled) prior to modeling. Environmental predictors were all aggregated to site level. We did not include site or year as fixed effects because environmental variables were measured at each site every year and we did not have enough replication to justify including them as random effects. To identify the best model among plausible alternatives, we compared nested models using “dredge” (*MuMIn*, Bartoń, 2020) and selected the model with the lowest AICc, with the constraints that *taxon* and linear *size*_*t*_ terms were always included, and quadratic terms were not included without their linear counterparts (see Appendix B.1 for a note on an alternative model selection approach we tried and rejected). For all best models, we visually checked model assumptions, plotted estimated marginal effects (from *ggeffects*, Lüdecke, 2018), and present *R* ^2^ or pseudo‐*R* ^2^ estimates of model fit (theoretical pseudo‐*R* ^2^ estimates for binomial models and delta estimates for negative binomial models; *MuMIn*, Bartoń, 2020). For binomial models, we calculated the area under a Receiver Operating Characteristic curve (AUC) as an estimate of model performance (where AUC = 1 indicates that the model perfectly classifies individuals, for example, as reproductive or not; *pROC*, Robin et al., 2011). Confidence intervals were calculated from standard errors using *ggeffects* (Lüdecke, 2018). When best‐supported models retained interaction terms (*taxon* × continuous predictor), we tested the significance of linear relationships for each taxon and then statistically compared taxon slopes using post‐hoc contrasts with Tukey's method to adjust for multiple comparisons (Lenth, 2020).

##### IPM construction

**FIGURE A5:**
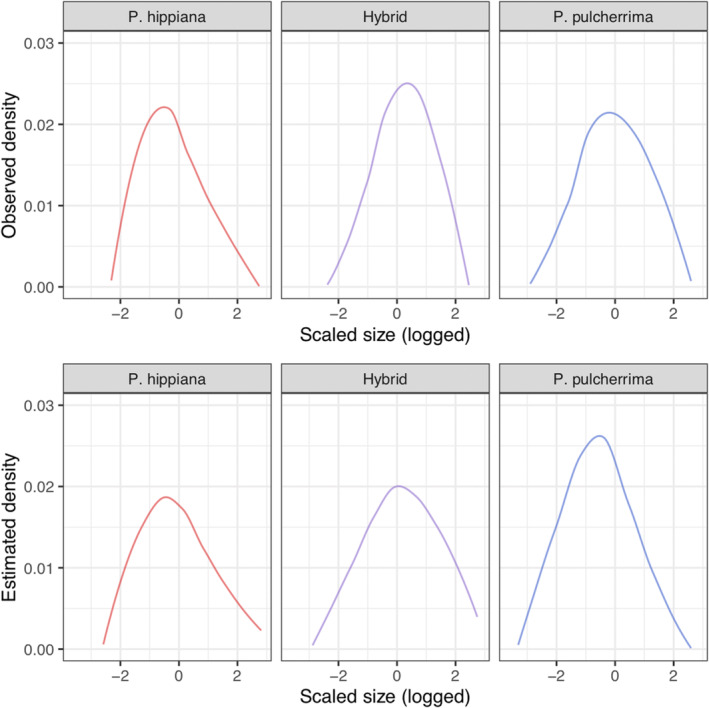
Stable stage distributions. Density based on observed abundances (top) versus from matrix decomposition from the IPM (based on the predicted densities from one example matrix per population per transition; bottom). All panels include a loess line for visualization. Seedlings are only slightly smaller than average scaled plant sizes and contribute to density peaks.

We parameterized IPMs separately for each taxon using the coefficients from the best‐supported vital rate models (see Appendix B.2 for details on IPM construction and analysis). We did not estimate lambdas for populations with fewer than 10 individuals due to insufficient data. We verified that our IPMs were generating generally reasonable estimates by comparing predicted to observed stable stage distributions for each taxon (Figure A5 in Appendix A).

##### Environmental responses

To assess the effects of environmental conditions on the population growth rates of each taxon, we estimated lambda for each population using their observed (site‐specific) environmental conditions in each year. We held density at the lowest observed for the taxon since species' responses to abiotic environmental variables are best described by their ability to grow from low densities with minimal intraspecific interactions (Birch, 1953; Louthan et al., 2015). Using the vital rate models, we generated 1000 sets of estimated coefficients for each vital rate by bootstrapping the plant size and environmental data. We bootstrapped these data using the Boot function in the *car* package (Fox & Weisberg, 2019) to resample data “from the joint distribution of the model and the response.” We then used each set of coefficients to estimate lambda across site‐specific temperature, snow cover duration, and elevation.

To explore how population growth rates will respond to variation in environmental drivers, we predicted lambdas of each taxon under combinations of environmental conditions using two scenarios (i.e., using each taxon's seedling/flower ratio from either 2018 to 2019 or 2019 to 2020 in the IPM). We explored 56 environmental combinations to capture the range of scaled environmental values we observed: 7 snow cover durations (every 0.5 between −2 and 1 scaled values), and 8 early summer temperatures (every 0.5 between −2 and 1.5 scaled values) at the study's median elevation (scaled value 0.28; high and low elevations produced qualitatively very similar results, and our study is not designed to model elevational responses due to its limited replications across elevation). We estimated each taxon's lambda (under each set of environmental conditions, in each scenario) using 100 of the previously generated bootstrapped sets of vital rate coefficients. For each combination of taxon, scenario, and environmental conditions, we averaged lambdas across the 100 bootstrap replicates to produce heatmaps showing how each taxon was predicted to respond under each combination of environmental variables.

## 3 RESULTS

### 3.1 Environmental conditions

**FIGURE A6:**
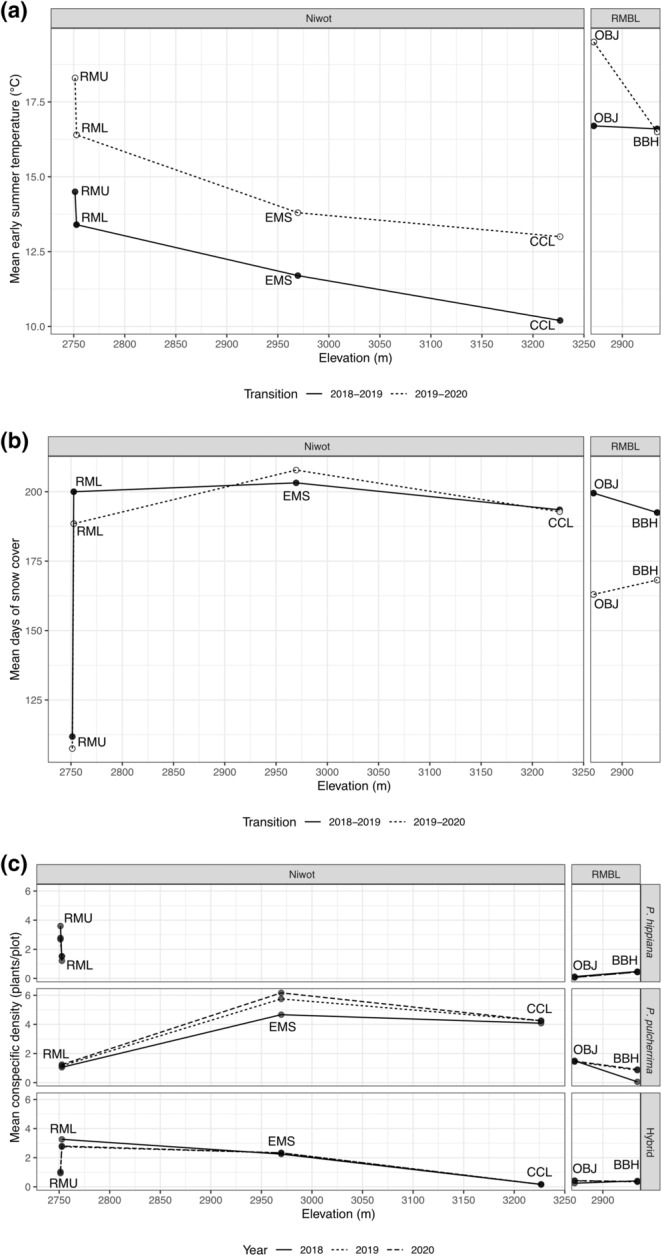
Changes in climate with elevation in study regions. Changes in summer temperature (a), duration of winter snow cover (b), and intraspecific density (c) with elevation. Points and labels denote sites, and transitions and years are indicated with solid or dashed lines. Lines connecting the sites are a visual aid to explore climate trends across elevation.

The summer (June to early July) of 2019 was, on average, ~2.4°C cooler than in 2020, but some sites deviated from this pattern (Figure A6a in Appendix A). Site‐specific temperatures decreased with increasing elevation (Figure A6a in Appendix A) and likely depended on (unmeasured) slope and aspect since the sites with the most exposed slopes also had the warmest early summer average temperatures (RMU, OBJ, BBH).

**FIGURE A7:**
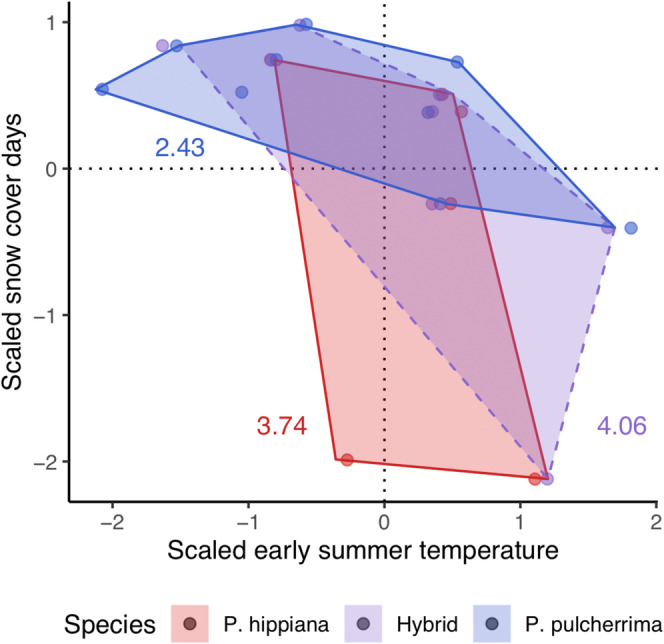
Breadth of climate experienced by focal taxa. Polygons show the breadth of environments each *Potentilla* taxon (colors) was exposed to across the study. Numbers are polygon areas, and points are sites in specific transitions, jittered for visibility. Although it is common in the study area, *P. pulcherrima* experienced the least variation in climate.

Sites had an average of 12 more days of snow cover in winter of 2018–2019 compared to 2019–2020 (Figure A6b in Appendix A). The magnitude of this difference varied substantially between the eastern and western slopes of the Continental Divide due to differences in regional precipitation trends (Figure A2 in Appendix A). The difference in snow cover duration was particularly dramatic at OBJ (western slope), which had ~37 more days of snow cover in 2018–2019 due to heavy snowfall and an avalanche (Figure A6b in Appendix A). In contrast, the duration of snow cover was almost identical, <5 days different, between the two winters at two of the four eastern slope sites (CCL, RMU; Figure A6b in Appendix A). Unlike summer temperature, the duration of snow cover did not vary consistently with elevation (Figure A6b in Appendix A). Focal taxa experienced similar magnitudes of climate variation across space and time (Figure A7 in Appendix A), although *P. pulcherrima* and the hybrid were exposed to a broader range of temperatures than *P. hippiana*, while *P. hippiana* and the hybrid encountered a broader range of winter snow cover duration than *P. pulcherrima*. Intraspecific density did not vary consistently with elevation or between regions (Figure A6c in Appendix A).

### 3.2 Vital rate models

**FIGURE 2:**
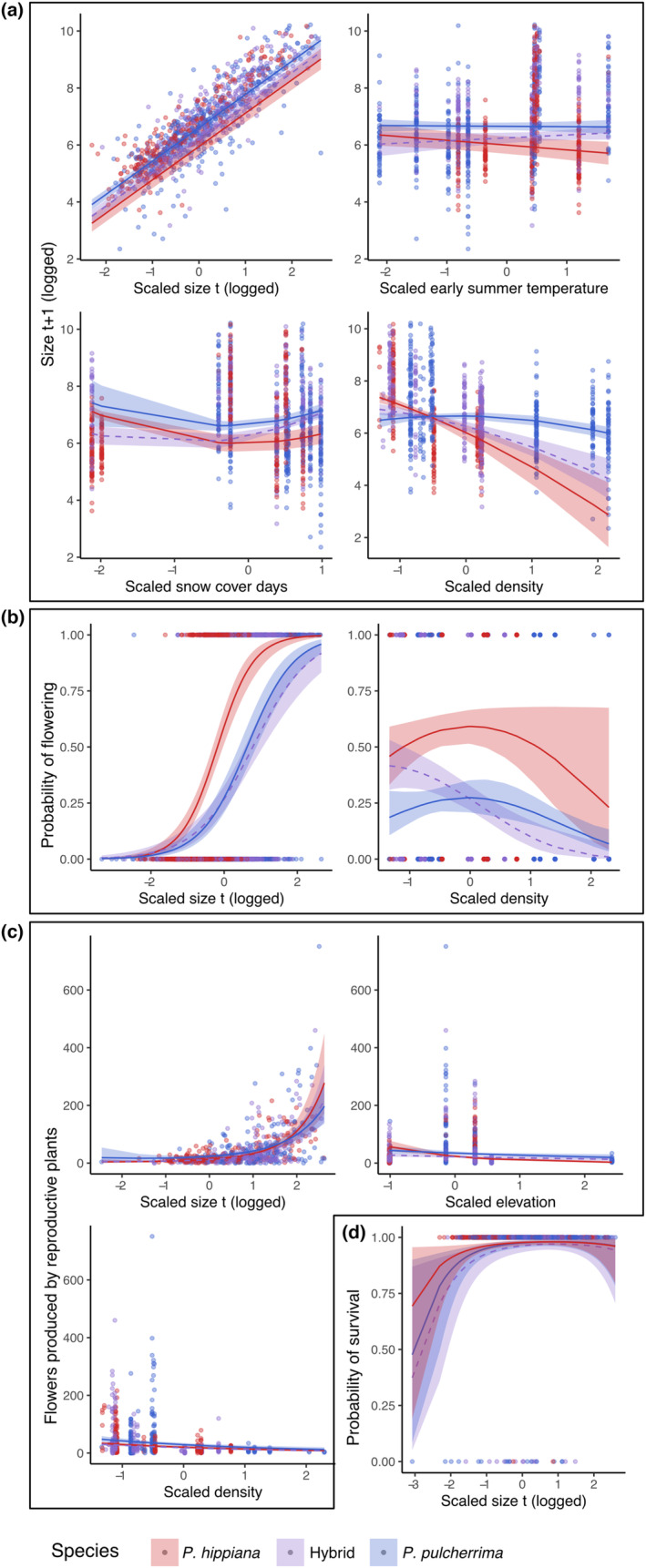
Marginal effects plots of the best vital rate models. Taxa are shown with colors (hybrids are purple dashed lines). Vital rates: (a) growth, modeled as size at *t* + 1, (b) probability of flowering, (c) flowers produced, (d) probability of survival. See Section 2 and Table 1 for model descriptions. Ribbons are 95% confidence intervals based on SE. Plots were produced with *ggeffects* (Lüdecke, 2018).

The best model for mean individual growth (*size*_*t*+1_) retained early summer temperature, duration of snow cover, and population density as environmental predictors as well as interactions between each environmental predictor and taxon (see Table 1 for model formulae). The model explained nearly 80% of variation in ${\text{size}}_{t+1}$ (*R* ^2^ = .78; Figure 2a), with the vast majority of variation in ${\text{size}}_{t+1}$ predicted by ${\text{size}}_{t}\;$(*R* ^2^ = .75 for a model with only ${\text{size}}_{t}\;$as a predictor). While some individuals grew and others shrank between surveys, large plants tended to remain large, and small plants remained relatively small across the study. For *P. pulcherrima*, ${\text{size}}_{t+1}$ had no significant relationship with any of the environmental predictors (*p* > .05 for each, Table A1 in Appendix A, Figure 2a). Across all three taxa, plant ${\text{size}}_{t+1}$ varied little with temperature (Figure 2a); however, the average ${\text{size}}_{t+1}$ of *P. hippiana* plants decreased slightly (though significantly) with warming, while hybrids showed an opposing tendency of slight (but not significant) increases in average ${\text{size}}_{t+1}$ with warming (Table A1 in Appendix A). Only hybrid ${\text{size}}_{t+1}$ increased significantly with duration of snow cover (Figure 2a, Table A1 in Appendix A). *Potentilla hippiana* and the hybrid were both larger at low densities (Figure 2a, Table A1 in Appendix A). Because *P. hippiana* and the hybrid were only found in relatively low densities at our study sites, we have greater certainty in predicting ${\text{size}}_{t+1}$ at low density (which we use for all population growth rate estimates) than high density for these two taxa.

The best model for the probability of flowering omitted elevation as a predictor but retained density as well as the *taxon* × *density* and *taxon* × *size*_*t*_ interaction terms (Table 1; pseudo‐*R* ^2^ = .51, AUC = 0.86). The probability of flowering increased at an accelerating rate with plant size for all focal taxa, while *P. hippiana* flowered at significantly smaller sizes than the hybrid (Figure 2b, Table A1 in Appendix A). Flowering probability had a significant negative linear relationship with density for the hybrid (Table A1 in Appendix A, Figure 2b) but peaked at intermediate densities for the parent taxa (Figure 2b).

Floral production by reproductive plants was predicted by elevation and density, as well as the *taxon* × *elevation* and *taxon* × *size*_*t*_ interaction terms (Table 1; pseudo‐*R* ^2^ = .36). Larger reproductive plants produced more flowers overall, though the relationship between size and floral production was significantly weaker in *P. pulcherrima* than in *P. hippiana* or the hybrid (Figure 2c, Table A1 in Appendix A). Floral production of both parent taxa decreased with elevation, especially for *P. hippiana*. The hybrid response was more moderate, particularly compared to *P. hippiana* (Table A1 in Appendix A). Floral production decreased with increasing population density (Figure 2c) and varied substantially across surveys and among taxa (1700–6000 per taxon per year). However, the number of seedlings remained uniformly low (0–7 per population per year), indicating that changes in reproductive output had little effect on short‐term seedling recruitment. Ratios of seedlings to flowers for all taxa were higher in the 2018–2019 transition, and on average > 3 × the 2019–2020 values (2018–2019 mean: 0.012; 2019–2020 mean: 0.0034).

For all three taxa, small plants had the lowest survival, with average‐sized plants reaching estimated survival probabilities >.95 (Figure 2d). *Potentilla hippiana* had the highest average survival probability while hybrids had the lowest (Figure 2d), though we could not statistically compare survival rates among taxa because the model estimating *P. hippiana* survival required us to use data from all three taxa (see Section 2). The pooled model used for *P. hippiana* explained little variation in survival (pseudo‐*R* ^2^ = .035) and classified individual survival only marginally better than random (AUC = 0.52) because size was a poor predictor of survival. Compared to the pooled model, the survival model used for *P. pulcherrima* and the hybrid performed better (pseudo‐*R* ^2^ = .21, AUC = 0.67) because it included a taxon term that best explained variation in survival rates.

### 3.3 Population growth rates

**FIGURE 3:**
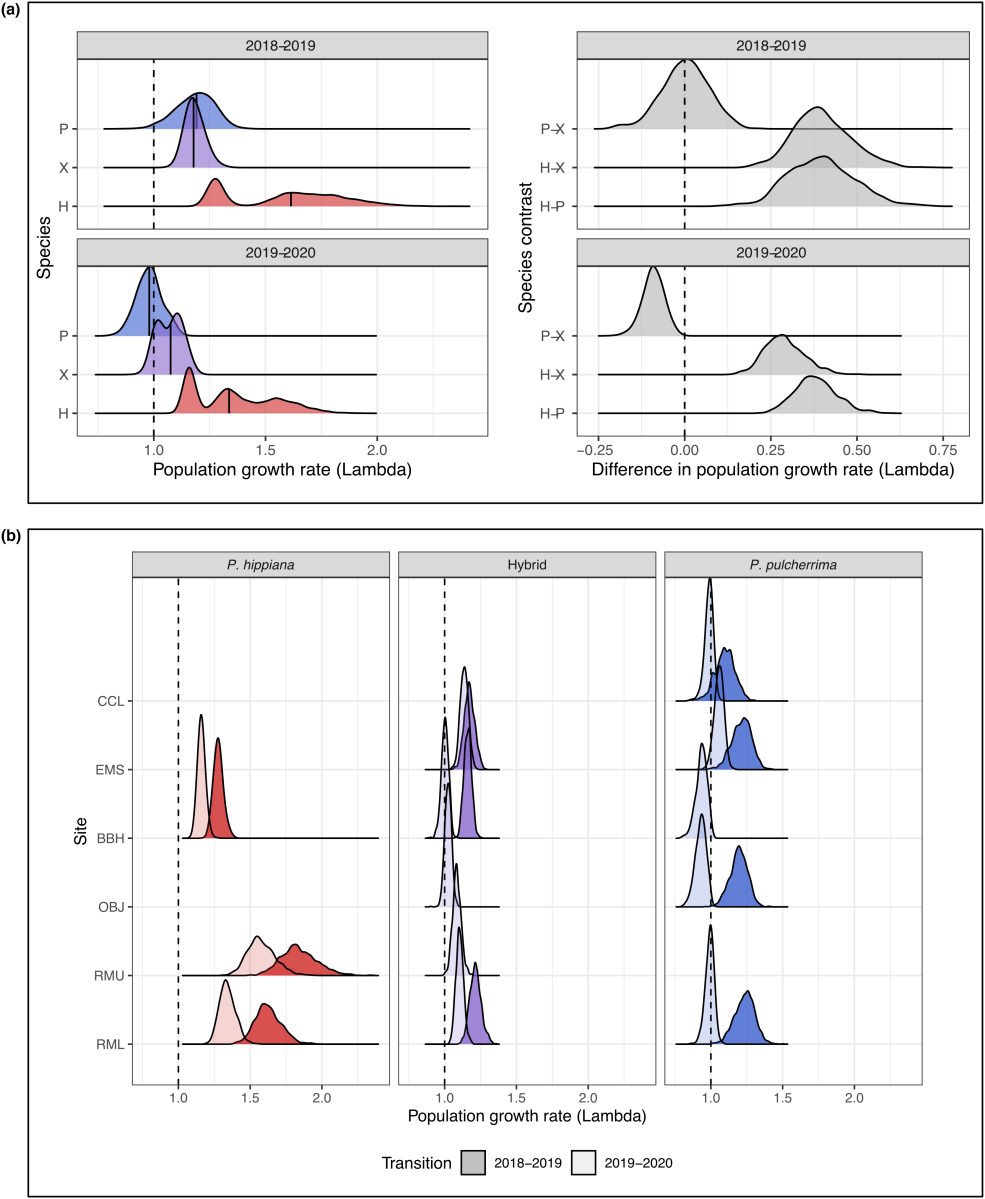
Probability density distributions of estimated population growth rates of each taxon across transitions. (a) Left: Probability density distributions of estimated population growth rates (lambdas) pooled across populations within taxa (solid black lines are median lambdas; dashed lines are the threshold for stable populations, lambda = 1). Taxa are indicated with colors and symbols (P = *P. pulcherrima*, X = hybrid, H = *P. hippiana*), and panels represent transitions. Lambda estimates for individual populations were derived from 1000 bootstraps of the underlying vital rate models and pooled by taxon for visualization. Right: densities of the differences in population growth rates between taxa (lambdas were pooled by taxon and the differences among taxa were calculated within each bootstrap replicate). For example, when P–X is negative (as in 2019–2020), the hybrid has higher population growth rates than *P. pulcherrima*. (b) Probability density distributions of estimated population growth rates for each population and transition. Transitions denoted with dark or light fill. Sites are ordered along the *y*‐axis from low (RML) to high (CCL) elevation. OBJ and BBH are west of the Continental Divide while all other sites are east of the Divide.

**FIGURE A8:**
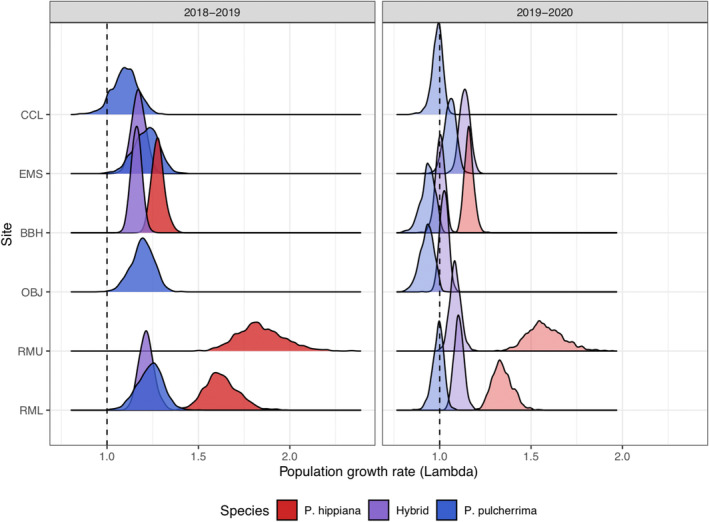
Probability densities of population growth rate estimates within sites and transitions. Note, this is the same data as Figure 3, reformatted for comparing populations within sites. Taxa are distinguished by colors, and transitions are shown with panels and opacity (dark or light fill). Lambda estimates are derived from 1000 bootstrap replicates of the underlying vital rate models. Sites are ordered along the *y*‐axis from low (RML) to high (CCL) elevation. OBJ and BBH are near RMBL (western slope of the Continental Divide) while all other sites are near Niwot (eastern slope of the Divide).

**FIGURE A9:**
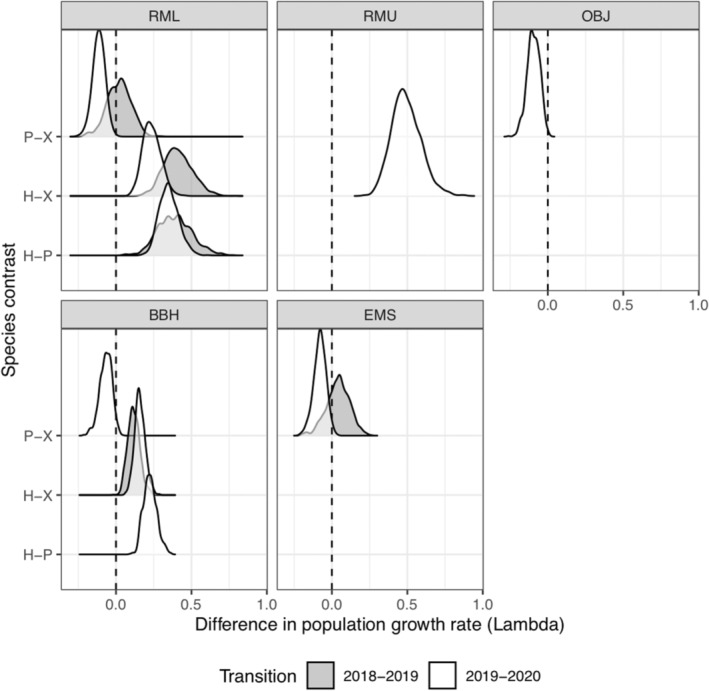
Densities of the differences in population growth rates between taxa within sites. Within each of 1000 bootstrap replicates, differences in lambda among co‐occurring taxa (within sites) were calculated. For example, when P–X is negative (as in 2019–2020 at BBH), the hybrid has higher population growth rates than *P. pulcherrima*. Sites that had co‐occurring taxa are ordered from lowest (RML) to highest (EMS) elevation.

Predicted population growth rates (lambda) ranged from 0.79 (catastrophic decline) to 2.35 (population boom) in our study (where stable populations occur at lambda = 1). All taxa, and nearly all individual populations, had higher lambdas in 2018–2019 than 2019–2020 (Figure 3). *Potentilla hippiana* had higher median lambdas than *P. pulcherrima* or the hybrid across the study period (Figure 3a). All *P. hippiana* populations had lambdas >1 throughout the study, but estimates ranged from moderate (at BBH) to extreme (at RMU) (Figure 3b), generating an overall distribution of *P. hippiana* population growth that was bimodal (Figure 3a). All *P. pulcherrima* populations were predicted to be either growing or stable under the cool, wet conditions of 2018–2019 but performed relatively poorly under drier, hotter conditions in 2019–2020, with lambdas mostly at or below replacement (slightly above replacement in EMS; Figure 3b). The hybrid maintained median lambdas at or above replacement throughout the study, indicating that the relative performance of the hybrid and *P. pulcherrima* shifted through time as *P. pulcherrima* populations fluctuated while hybrid population growth rates remained relatively consistent (Figure 3a, Figures A8 and A9 in Appendix A).

**FIGURE 4:**
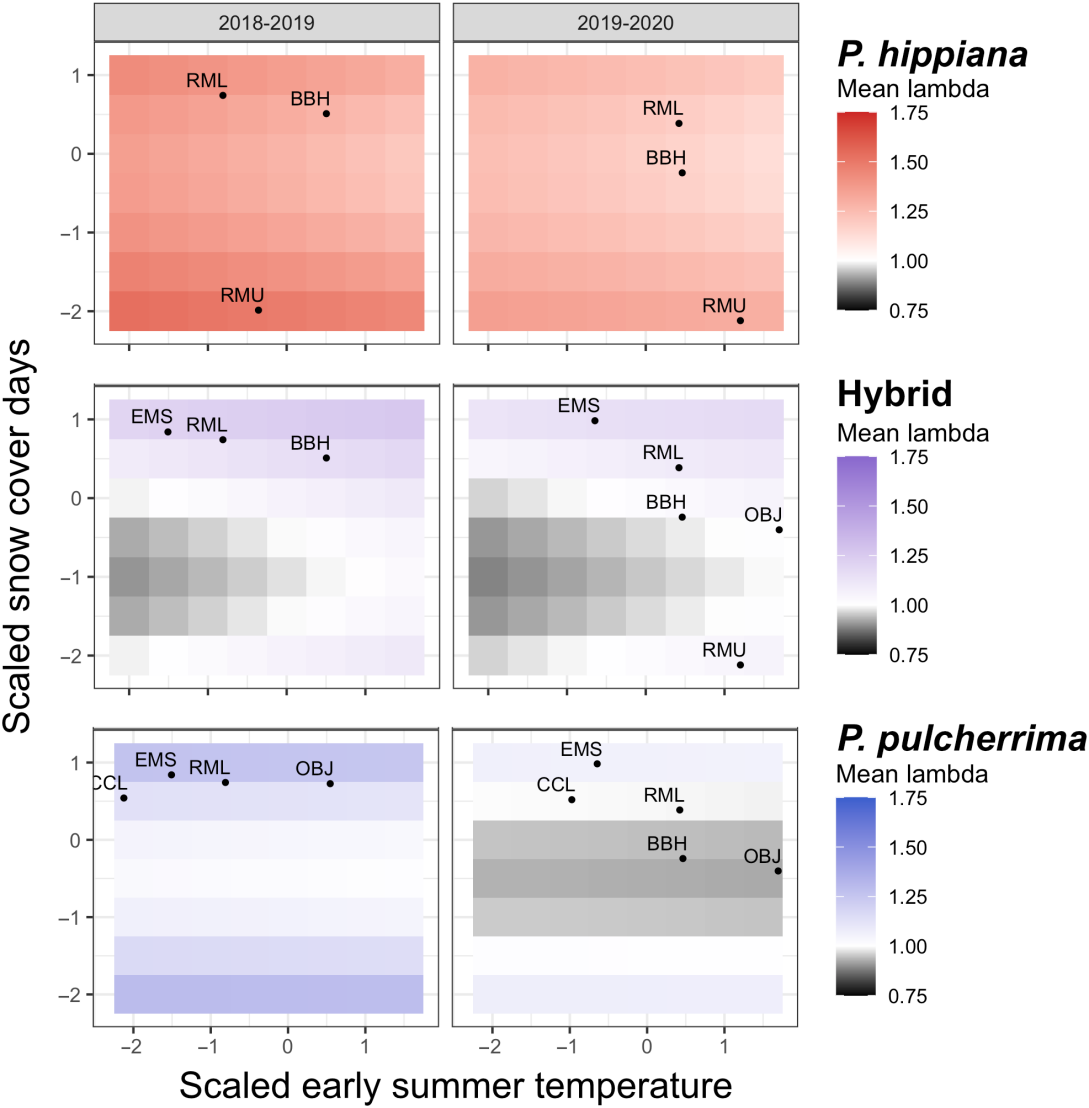
Response of predicted population growth rates to new climates. Colors are mean predicted population growth rates (lambdas) of focal taxa under combinations of early summer temperature and duration of winter snow cover, at the study's median elevation (0.28 scaled). Colors delineate shrinking (lambda < 1), stable (lambda = 1), and growing (lambda > 1) populations. Within each taxon and scenario (IPMs using seedling/flower ratios from 2018 to 2019 or 2019 to 2020), lambdas for each environmental combination are averages of estimates from 100 bootstrap replicates (see Section 2). Points mark observed climates in each site and transition.

For both parent taxa, lambdas were insensitive to increasing early summer temperature and greatest at the extremes (low and high) of snow cover duration (Figure 4). Lambda of *P. pulcherrima* was particularly depressed at moderate snow cover duration when lambdas were modeled using 2019–2020 values of seedling/flower ratios (right panel, Figure 4), since the average seedling/flower ratio for *P. pulcherrima* populations was 7‐fold lower in 2019–2020 compared to 2018–2019. Predicted population growth of the hybrid increased with both temperature and snow cover duration, with the highest temperatures leading to stable populations even under unfavorable (mid‐low) snow cover duration (Figure 4).

## 4 DISCUSSION

This study provides a rare comparison of the demography of hybridizing plant taxa across a range of environmental conditions, providing insight into how phenotypic variation generated through hybridization may impact plant demographic responses to changing environments. We combined demographic monitoring of multiple natural populations with measures of site‐specific climate variables to quantify how vital rates and patterns of population growth change over space and time and among closely related taxa. Based on the current distributions of the parent taxa, we had expected that *P. hippiana* populations would have positive growth rates in dry conditions and be less tolerant of other environments, while *P. pulcherrima* would exhibit positive or stable population growth across a broader range of conditions. Instead, we found that *P. hippiana* exhibited positive predicted population growth rates under all conditions that it experienced in the subalpine, while *P. pulcherrima* performance was highly dependent on site and survey year, with most populations predicted to shrink under the relatively hot and dry (i.e., shorter snow cover) conditions that occurred between 2019 and 2020 (Figures 3 and 4). Taken together, these results suggest that the spatial distributions of parent taxa may be lagging behind the rate of environmental change to drier and warmer conditions, as the taxon that showed the highest population growth rates (*P. hippiana*) across all conditions is currently the least abundant. We anticipate that *Potentilla hippiana* should become more prevalent over time, while *P. pulcherrima* populations decline, as conditions become increasingly warm and dry. The predicted performance of hybrids was not consistently better nor worse than parents as a whole and instead was contingent on the environment and the parent taxon to which they were compared (Figures 3 and 4, Figures A8 and A9 in Appendix A). Consequently, our results suggest that demographic trajectories of even closely related, co‐occurring taxa can be quite different, potentially leading to shifts in their relative abundances as climates continue to change.

### 4.1 Population growth rates of parent and hybrid taxa

Slow population turnover in *Potentilla* could lead to demographic lags in response to rapidly changing environments. We observed very low mortality and, although the populations in our study produced numerous flowers, recruitment remained low to absent (Figure 2). This life history, dominated by individual growth and survival rather than offspring production, typifies many long‐lived plants (Kuss et al., 2008; Sulis et al., 2018; Ulrey et al., 2016) and can generate mismatches between species' current distributions and their demographic trajectories (Pagel et al., 2020). We found that *P. hippiana* was the only taxon predicted to maintain positive growth rates in all of its populations throughout the study (Figure 3b), yet it is presently much less common than *P. pulcherrima* in the study area. *Potentilla pulcherrima*'s comparatively poor performance and its sensitivity to warm, dry conditions (Figure 3) imply that its current high abundance may be a legacy of a cooler, wetter past.

Theories of hybrid vigor (or, conversely, hybrid breakdown) predict that hybrids should consistently perform better (worse) than parental taxa based on interactions between parent genomes, regardless of the environment (East, 1936; Mayr, 1942; Simon et al., 2018). However, the hybrid populations included in our study had lower predicted population growth rates than *P. hippiana* overall (inconsistent with hybrid vigor) but do not appear to be uniformly unfit (inconsistent with hybrid breakdown; Figure 3). Studies in mulberry (*Morus*; Burgess & Husband, 2006) and phlox (*Phlox*; Levin & Schmidt, 1985) also found no evidence of hybrid vigor or breakdown, reporting hybrid vital rates that were comparable to one or both parent taxa across habitats. These patterns suggest that hybrid success is context‐dependent, varying with the environment and/or the genetic identity of the hybrids (following Campbell & Waser, 2001). Hybrids had slightly lower median lambdas than *P. pulcherrima* across the cooler, wetter period (2018–2019) but intermediate lambdas relative to the parental taxa under more typical (2019–2020) conditions (Figure 3). A similar pattern of environmental‐dependent hybrid performance was found in spruce (*Picea*): hybrids between high‐ and low‐elevation spruce taxa were suboptimal at the elevations of each parent, but outperformed the parents at intermediate elevations, where hybrids grew taller than one parent and set buds more quickly than the other (De La Torre et al., 2014).

**FIGURE A10:**
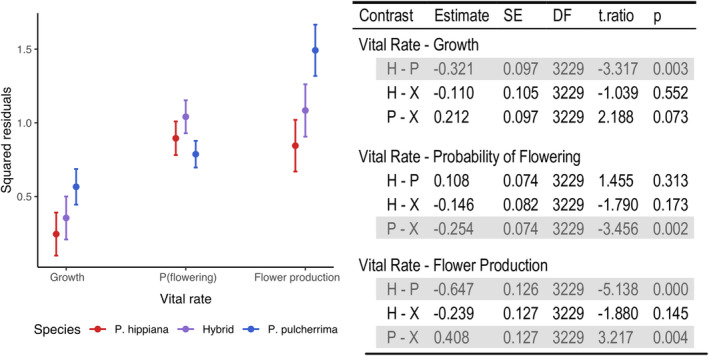
Comparing predictability of hybrid and parent taxa. (Left) Points show estimated marginal mean squared residuals for each taxon (color) in each vital rate model. Lines indicate 95% confidence intervals. The survival model is not shown because *P. hippiana* survival was estimated from a global model without a taxon term (see Section 2). We tested whether squared residuals of hybrids were higher than parents for each vital rate. If hybrid vital rates were more poorly predicted than the parent taxa, it would suggest that hybrids are a more heterogeneous class. (Right) Pairwise contrasts of residuals by vital rate and taxon (H = *P. hippiana*, X = hybrid, P = *P. pulcherrima*). Significant contrasts are highlighted in gray, and *p*‐values are adjusted using Tukey's adjustment for multiple comparisons, using *emmeans* (Lenth, 2020).

If hybrids continuously interbreed with parents and/or each other, then “hybrids” are a heterogeneous group that includes several classes (first‐generation crosses, backcrosses with parent lineages, and later‐generation hybrids) that may vary in fitness and environmental affinities (Arnold, 1997). Previous work has shown that hybrid performance can depend on which taxon is the maternal plant (Burgess & Husband, 2006) and that hybrid vigor in first‐generation hybrids can give way to selection against unfit hybrids in later generations as recombination exposes recessive incompatibilities between parent genomes (Johansen‐Morris & Latta, 2006). In our study, hybrids did not seem to be a more heterogeneous group than the parent taxa, based on the residuals from vital rate models (Figure A10 in Appendix A). Preliminary sequencing analyses indicate that hybrids cluster with parent taxa within sites, especially at RMBL sites (Figure A1 in Appendix A). This pattern could indicate either within‐site gene flow between hybrid and parent taxa (and, therefore, a wide range of hybrid ancestries) or recent hybrid origins (not necessarily ongoing gene flow). More detailed genetic analyses would be needed to characterize the range of ancestries of the *Potentilla* hybrids at these sites.

### 4.2 Climate impacts on population growth

Several studies of montane plant species have found that population growth rates increase with elevation (Angert, 2009; Krushelnycky et al., 2013). However, the *Potentilla* taxa in our study did not show consistent changes in population growth rates across elevations (Figure 3b; similar to Oldfather & Ackerly, 2019; Pollnac et al., 2014). In the mountainous areas where *Potentilla* populations occur, it is likely that local environmental conditions are heavily influenced by local topography, which creates a complex mosaic of temperature and moisture across elevations rather than consistent linear trends (see Keller et al., 2005; Oldfather et al., 2020). In our study, predicted population growth rates of the parent taxa declined sharply with observed warming (e.g., as RMU and RML warmed 3.0 and 3.8°C, respectively, between summer of 2019 and 2020; Figure A6 in Appendix A). However, our population growth estimates across new environmental combinations suggest that snow cover duration has a larger net impact on lambda, with intermediate snow cover duration associated with reduced lambda. It is possible that the additional moisture and insulation that occurs in long snow cover years (e.g., Litaor et al., 2008) offset the negative impacts of shorter growing season, and that years with shorter periods of snow cover are also warmer years and thus less prone to harmful spring frosts. Our results join a larger body of work on montane plant taxa (including *P. pulcherrima*) documenting decreased survival and reproduction under warm, dry conditions (Campbell, 2019; Reynolds, 1984; Stinson, 2005), potentially leading to local extinctions of some populations under the climate combinations expected in the future (Panetta et al., 2018).

Over the last century, the Colorado Rocky Mountains have experienced increasing temperatures and substantial decreases in snowpack (McGuire et al., 2012; Pederson et al., 2011; Qin et al., 2020), and these climate trends are projected to continue (Adam et al., 2009). Our results suggest that these climate trends will reduce population growth rates for all our focal taxa (Figures 3 and 4), although *P. hippiana*—the taxon that currently occupies the driest, hottest environments—may maintain positive population growth rates (at least in the near term). Expected climate change will have particularly negative effects on *P. pulcherrima*, whose populations were already pushed below replacement in 2020 and are particularly sensitive to reduced snow cover duration (Figure 4). Median lambda for *P. pulcherrima* dropped 18% from 2019 to 2020, so continued exposure to warm, dry conditions could cause these populations to rapidly dwindle, unless the high site‐to‐site heterogeneity in climate exposure within mountain habitats provides microrefugia that buffer the rate of overall species decline (Figure A6 in Appendix A; Oldfather & Ackerly, 2019).

### 4.3 Caveats and future directions

Despite the breadth of sampling across sites and among taxa, the short temporal scale of our study provides only a snapshot of the demographic trends of hybridizing taxa under changing conditions. If large, infrequent mortality or recruitment events are a critical component of *Potentilla* long‐term demography, our data may not predict *Potentilla* climate change responses over longer time periods. Population responses to inter‐annual climate variation may not match demographic patterns across broader latitudinal gradients (Peterson et al., 2021); hence, our inferences about *Potentilla* demography are constrained to populations on either side of the Continental Divide in the Colorado Rockies. We measured snow cover duration and discussed short snow cover years as ‘dry’ due to its effects on growing season soil moisture, but growing season precipitation is another axis of moisture that would be valuable to explore in future work. Our study cannot rule out the possibility that variation in other nonclimatic factors (e.g., pollination, herbivory) across the study period influenced population growth rates. Studies over longer periods of time, or in other parts of *Potentilla*'s geographic range, are needed to understand the extent to which our results predict how these taxa will respond to ongoing climate change. More generally, additional research is needed that uses comparative demographic approaches to understand the trajectories of diverse genotypes experiencing rapid and unprecedented environmental change. Coupling such observational studies with experiments that tease apart the genetic and environmental components of performance would provide additional insight into hybrid adaptation and evolution.

## AUTHOR CONTRIBUTIONS

**Kelly A. Carscadden:** Conceptualization (lead); data curation (lead); formal analysis (lead); funding acquisition (lead); investigation (lead); methodology (lead); project administration (lead); resources (lead); supervision (lead); visualization (lead); writing – original draft (lead); writing – review and editing (lead). **Daniel F. Doak:** Conceptualization (supporting); formal analysis (supporting); methodology (supporting); resources (supporting); supervision (supporting); writing – original draft (supporting); writing – review and editing (supporting). **Meagan F. Oldfather:** Formal analysis (supporting); investigation (supporting); methodology (supporting); writing – original draft (supporting); writing – review and editing (supporting). **Nancy C. Emery:** Conceptualization (supporting); funding acquisition (supporting); investigation (supporting); methodology (supporting); project administration (supporting); resources (supporting); supervision (supporting); writing – original draft (supporting); writing – review and editing (supporting).

## Data Availability

Data will be available through the Niwot LTER Environmental Data Initiative (nwt.lternet.edu/data‐catalog) upon publication. DOI: https://doi.org/10.6073/pasta/4f5913f9095b74945700d1dae93c0f16

## APPENDIX A Figures and Tables

**TABLE A1:** Vital rate model summaries.

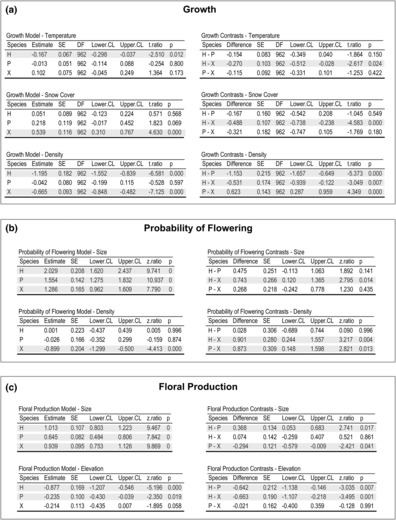

*Note*: Summaries of best vital rate models, corresponding to Figure 2. Left column: Estimated marginal means of *linear* trends in best vital rate models containing interaction terms. Right column: Contrasts of slopes between taxa. H = *P. hippiana*, P = *P. pulcherrima*, and X = hybrid. Standard error, degrees of freedom (for the Gaussian model of mean growth), (a) and 95% confidence intervals are shown. For generalized linear models (b, c), confidence intervals are asymptotic, and *z*‐ratios are reported instead of *t*‐ratios. Significant results are highlighted with gray. Table produced with *kableExtra* (Zhu, 2020).

## APPENDIX B Model details

### B.1 VITAL RATE MODEL SELECTION

We selected the best model for each vital rate using “dredge” (see Section 2 in main text). We compared dredge results to Lasso, a popular feature selection technique that shrinks coefficients of unnecessary parameters to zero (Friedman et al., 2010), for growth and reproduction (which began with more complex models than the other vital rates). For our models, Lasso was very sensitive to the penalty parameter chosen and selected either much simpler or more complex models (vs. dredge with AICc) depending on the penalty. Therefore, we present only results from dredge and AICc in the main text, since this approach favored biologically reasonable models of intermediate complexity.

### B.2 IPM CONSTRUCTION

Our IPMs incorporate the combined effects of growth, survival, and reproduction to predict population growth rates. IPMs are based on a kernel that predicts size‐specific transitions across time steps (Metcalf et al., 2012):

$$n\left(y,t+1\right)=\int_{L}^{U}K\left(y,x\right)n\left(x,t\right)\mathrm{dx}=\int_{L}^{U}\left[P\left(y,x\right)+F\left(y,x\right)\right]n\left(x,t\right)\mathrm{dx}$$

Here, the numbers of individuals of each size and, *K*, the kernel is integrated between upper and lower size boundaries to predict the number of individuals of size *u* at *t* + 1, *n*(*y*, *t* + 1). The kernel is composed of two subkernels, *P* and *F*, where *P* represents transitions attributable to survival and growth, and the *F* kernel describes per‐capita contributions to reproduction. Specifically, *P* is derived from the product of the Growth and Survival vital rate models described in Table 1, while F is the product of the Floral production and Recruitment models (Table 1). As described in the main text, the best‐supported vital rate models other than Recruitment are context‐specific, and thus we parameterize the IPM kernel for different values of the driver variables (elevation, taxon, density, etc.).

While IPMs are theoretically descriptions of continuous state variable (size in our case) changes, in practice their analysis relies on discretizing the continuous subkernels into transition probabilities of plants into different size bins. We defined the size bounds of the IPM as 1.1× the *scaled* maximum and minimum (negative) sizes to allow individuals slightly beyond the size ranges observed. We partitioned this size range into 75 evenly sized bins (tests with more bins yielded similar model predictions). We determined the weighted median size of each bin from the empirical probability density function of sizes at time *t* (Doak et al., 2021), using data across all sites (with that taxon) and years and evaluated each vital rate model at these discrete size “mesh points” to parameterize the IPM. Predicted sizes at *t +* 1 (from the growth model) were scaled, to be consistent with the scaled sizes that are the basis of the size classes in the IPM. For each starting size, we used the mean scaled predicted size and the standard deviation of scaled residuals from the growth model (to account for variance in growth) to define a cumulative density function. The difference in the cumulative density function between bin edges provides the growth probabilities for the individuals in each bin (Morris & Doak, 2002; Peterson et al., 2021). We renormalized the sum of these differences to 1 to prevent any individuals from being evicted from the model (Williams et al., 2012). We multiplied the predicted survival and growth probabilities to generate a matrix of probabilities for transitions among size bins. New seedlings were added as a separate class. Since there were relatively few seedlings, we estimated their survival based on all small conspecific plants (smaller than or equal to average seedling size). Survival of *P. hippiana* seedlings was estimated from the survival of all plants (across taxa) less than or equal to average *P. hippiana* seedling size, since we did not observe any mortality of small *P. hippiana* plants during the study. For each taxon, we defined the cumulative density function of new seedling sizes using the mean and standard deviation of seedling sizes for that taxon across all years and sites, and we found normalized growth probabilities and transition probabilities as above. We included the formation of new individuals in the matrix by multiplying the predicted reproduction (predicted probability of reproduction X predicted number of flowers produced if reproducing) by the number of seedlings per flower. Recall that seedling/flower ratios were calculated per taxon per transition (see Section 2). Annual population growth rate (lambda) is the dominant eigenvalue of this final matrix (the combination of the reproduction and growth/survival matrices).
